# The efficacy and safety of left atrial low‐voltage area guided ablation for recurrence prevention compared to pulmonary vein isolation alone in patients with persistent atrial fibrillation trial: Design and rationale

**DOI:** 10.1002/clc.23677

**Published:** 2021-07-22

**Authors:** Akihiro Sunaga, Masaharu Masuda, Koichi Inoue, Nobuaki Tanaka, Tetsuya Watanabe, Yoshio Furukawa, Yasuyuki Egami, Akio Hirata, Nobuhiko Makino, Hitoshi Minamiguchi, Takafumi Oka, Tomoko Minamisaka, Toshihiro Takeda, Tomomi Yamada, Tetsuhisa Kitamura, Hirota Kida, Bolrathanak Oeun, Taiki Sato, Yohei Sotomi, Tomoharu Dohi, Katsuki Okada, Shinichiro Suna, Hiroya Mizuno, Daisaku Nakatani, Shungo Hikoso, Yasushi Sakata, OCVC‐SUPPRESS‐AF investigators

**Affiliations:** ^1^ Department of Cardiovascular Medicine Osaka University Graduate School of Medicine Suita Japan; ^2^ Cardiovascular Center Kansai Rosai Hospital Amagasaki Japan; ^3^ Cardiovascular Center Sakurabashi‐Watanabe Hospital Osaka Japan; ^4^ Cardiovascular Division National Hospital Organization Osaka National Hospital Osaka Japan; ^5^ Division of Cardiology Osaka General Medical Center Osaka Japan; ^6^ Division of Cardiology Osaka Rosai Hospital Sakai Japan; ^7^ Cardiovascular Division Osaka Police Hospital Osaka Japan; ^8^ Department of Cardiovascular Medicine Yao Municipal Hospital Yao Japan; ^9^ Department of Medical Informatics Osaka University Graduate School of Medicine Suita Japan; ^10^ Department of Medical Innovation Osaka University Hospital Suita Japan; ^11^ Department of Environmental Medicine and Population Sciences, Department of Social and Environmental Medicine Osaka University Graduate School of Medicine Suita Japan

**Keywords:** atrial fibrillation, catheter ablation, low‐voltage area guided ablation, randomized control trial, substrate modification, voltage mapping

## Abstract

Recurrence rates of atrial fibrillation (AF) after pulmonary vein isolation (PVI) are higher in patients with a left atrial low‐voltage area (LVA) than those without. However, the efficacy of LVA guided ablation is still unknown. The purpose of this study—the Efficacy and Safety of Left Atrial Low‐voltage Area Guided Ablation for Recurrence Prevention Compared to Pulmonary Vein Isolation Alone in Patients with Persistent Atrial Fibrillation trial (SUPPRESS‐AF trial)—is to elucidate whether LVA guided ablation in addition to PVI is superior to PVI alone in patients with persistent AF. The Osaka Cardiovascular Conference will conduct a multicenter, randomized, open‐label trial aiming to examine whether LVA guided ablation in addition to PVI is superior to PVI alone in patients with persistent AF and LVAs. The primary outcome is the recurrence of AF documented by scheduled or symptom‐driven electrocardiography (ECG) during the 1 year follow‐up period after the index ablation. The key secondary endpoints include all‐cause death, symptomatic stroke, bleeding events, and other complications related to the procedure. A total of 340 patients with an LVA will be enrolled and followed up to 1 year. The SUPPRESS‐AF trial is a randomized controlled trial designed to assess whether LVA guided ablation in addition to PVI is superior to PVI alone for patients with persistent AF and LVAs detected while undergoing their first catheter ablation.

## 1 INTRODUCTION

For patients with paroxysmal atrial fibrillation (AF), pulmonary vein isolation (PVI) to eliminate ectopic beats is the standard approach.^1^, ^2^ For patients with persistent AF, additional ablation after PVI has been considered necessary because persistent AF is usually accompanied by more complex triggers and a more extensive arrhythmogenic substrate.^3^ However, the efficacy and necessity of additional substrate modifications after PVI in patients with persistent AF remains controversial.

Recurrence rates of AF are higher in patients with left atrial low‐voltage areas (LVAs) than in those without.^4^ LVAs may represent fibrosis, as depicted by delayed‐enhanced magnetic resonance imaging,^5^ and are arrhythmogenic.^6^ Therefore, LVA guided ablation can be a tailored to eliminate the LVA arrhythmic substrate. Although several reports on LVA guided ablation have appeared, a number of these were not randomized controlled trials.^7^, ^8^, ^9^, ^10^ Although the superiority of additional complex fractionated atrial electrogram ablation or linear ablation after PVI was not demonstrated by the STAR AF II trial,^11^ some studies have made them a control group.^12^, ^13^, ^14^ The number of patients with an LVA in these studies is small because of their low prevalence (10%) in patients with paroxysmal AF^15^ and in patients with persistent AF (35%).^7^ The number of patients with an LVA in these studies was only 35–159.^7^, ^8^, ^9^, ^10^, ^12^, ^13^, ^14^, ^15^ To determine whether LVA guided ablation in addition to PVI is superior to PVI alone, the standard approach for AF, a sufficient number of cases with LVAs should be used with PVI alone as a control group.

In this multicenter, randomized trial, we aimed to elucidate whether LVA guided ablation in addition to PVI is superior to PVI with respect to the maintenance of sinus rhythm in patients with persistent AF with a sufficient sample size. This trial will compare the efficacy of LVA ablation in addition to PVI with PVI alone in patients with persistent AF and LVAs.

## 2 METHODS

### 2.1 Objective

The SUPPRESS‐AF trial will examine whether LVA guided ablation in addition to PVI is superior to PVI alone with respect to the recurrence of AF documented by electrocardiography during the 1 year follow‐up period after the ablation procedure.

### 2.2 Study design

**FIGURE 1:**
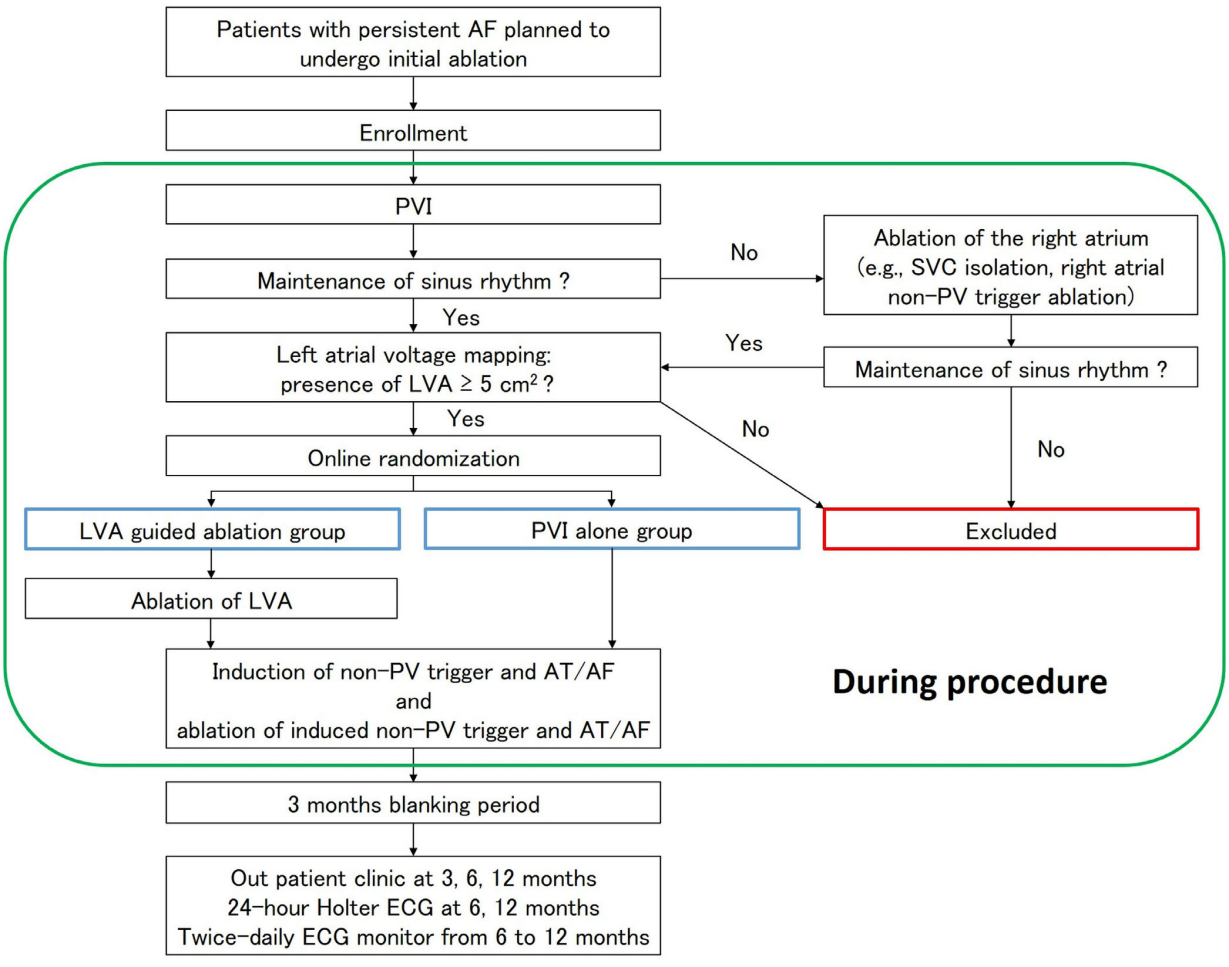
Study design. AF, atrial fibrillation; AT, atrial tachycardia; ECG, electrocardiogram; LVA, low‐voltage area; PVI, pulmonary vein isolation; PV, pulmonary vein; SVC, superior vena cava

The SUPPRESS‐AF trial is a prospective, multicenter, randomized, open‐label trial in which patients with persistent AF will undergo a catheter ablation procedure. The design of the SUPPRESS‐AF trial is summarized in Figure 1. After providing informed consent at each hospital, if operators confirm the presence of LVA(s) ≥ 5 cm^2^ during the procedure, patients will be randomized to either LVA guided ablation in addition to PVI or PVI alone. Patients who have given consent but do not have an LVA will be dropped out without allocation. Randomization will be performed electronically by entering patient information into an automatic data collection system via a secure Internet connection. Institutions will be the only adjustment factor considered in dynamic allocation to avoid bias. This study has been registered at UMIN‐CTR (UMIN000035940) and has been approved by the institutional review board. All patients provided written informed consent for their participation in this study. Clinical trial registration: UMIN‐CTR (UMIN000021831).

### 2.3 Eligibility criteria

The study population will consist of patients with persistent AF undergoing a first‐time ablation procedure. Persistent AF is defined as a sustained episode lasting ≥7 days at enrollment. Inclusion and exclusion criteria are summarized in Table 1. If patients do not have LVAs after consenting to the study, they will be excluded. A signed ethics committee/institutional review board‐approved informed consent form will be obtained from each patient prior to any trial‐related procedure.

**TABLE 1:** Eligibility criteria

Inclusion criteria for enrollment
Patients undergoing a first‐time ablation procedure for persistent atrial fibrillation
Exclusion criteria for enrollment
Patients with left atrial diameter ≥ 55 mm Patients with prior cardiac surgery Patients with valvular atrial fibrillation Patients receiving dialysis Patients aged <20 years Pregnant patients Patients with a history of stroke, transient ischemic attack, or systemic embolism within the past 6 months Patients who are not considered to be suitable candidates by the physician
Inclusion criteria for allocation
Patients with left atrial low‐voltage area
Exclusion criteria for allocation
Patients withdraw their consent Patients who are not considered to be suitable candidates by the physician

### 2.4 Procedures

Anti‐arrhythmic drugs will be discontinued for at least five half‐lives before procedures; amiodarone will be discontinued for at least 60 days. The physician will have the choice of using conscious sedation, deep sedation, or general anesthesia during procedures. Transseptal access to the left atrium will be used. Following the procedure, heparin will be administered to maintain an activated clotting time of >300 sec. Multipolar catheters (8 poles minimum) will be positioned in the coronary sinus and the right atrium, and multipolar catheters (10 poles minimum) will be used for mapping and PVI confirmation. Thermocool SmartTouch (Biosense‐Webster Inc., Diamond Bar CA, USA), an ablation catheter sensing contact force, will be used for ablation and mapping. All procedures will be guided using CARTO3 system (Biosense‐Webster Inc.) which is a 3‐dimensional cardiac mapping system. Anticoagulation therapy will be administered for at least 3 weeks before catheter ablation and 3 months after catheter ablation. After that time, it will be continued or stopped at the physician's discretion.

#### 2.4.1 Circumferential pulmonary vein isolation

All patients will undergo an initial ipsilateral circumferential PVI. Whether to perform cardioversion before PVI will be left to the discretion of the physicians. An esophageal temperature probe will be used for continuous real‐time monitoring during applications. A Visitag module (Biosense Webster Inc.), which is a real‐time automated display of applications, will be used with settings as follows: catheter stability ≤2 mm for 5 s, contact force ≥5 g (time ≥ 25%), tag size 4 mm in diameter. All radiofrequency applications will be based on the ablation index (AI). Radiofrequency will be delivered until an AI of ≥425 at the anterior wall and ≥ 375 at the posterior wall is achieved. When delivering radiofrequency around the esophagus, AI of ≥300 will be targeted, but target AI will be left to the discretion of the physicians in order to prevent complications. The maximum interlesion distance will be ≤4 mm.^16^ The standard ablation settings will consist of an upper‐temperature limit of 45°C, a radiofrequency power of 30–40 W, and a catheter irrigation flow rate of 17–30 ml/min. The radiofrequency power will be reduced to 25 W according to esophageal temperature. The success of PVI will be defined as the achievement of a bidirectional block in all PVs. The success of PVI will be reconfirmed at the end of the procedure, a minimum of 20 min after the initial success of PVI. If conduction in the LA and/or PV reappear, reisolation will be performed. Intravenous administration of adenosine triphosphate may be used to detect unablated foci at the discretion of the physician to confirm persistent PVI. If ATP reveals dormant conduction at sites in which the earliest activation is transiently observed on the circular mapping catheter, these sites will undergo further ablation, with confirmation by ATP infusion.

#### 2.4.2 Left atrial voltage mapping

After PVI, a detailed left atrial bipolar voltage mapping will be performed with pacing at a rate of 100 ppm on the high right atrium. If AF persists after PVI, electrical cardioversion will be used to restore the sinus rhythm. If sinus rhythm is not maintained, ablation of the right atrium (e.g., superior vena cava isolation, right atrial non‐PV trigger ablation) will be performed. The settings for ablation of the right atrium will be left to the physicians. If sinus rhythm is not obtained after ablation of the right atrium, the patient will be excluded. Ablation of the LA in addition to PVI to maintain sinus rhythm will not be performed to avoid any effect on LVAs. For left atrial bipolar voltage mapping, a multipolar 1 mm electrode catheter with a magnetic sensor will be used (Pentaray or Lasso Nav, Biosense‐Webster Inc., Diamond Bar CA, USA). The point acquisition will be performed with a CONFIDENCE® module (Biosense‐Webster) with the following settings: cycle length range ± 30 msec, local activation time stability 3 msec, position stability 2 mm, density 1 mm, and tissue proximity index off. Fast anatomical mapping volume will be 17 on a scale of 1–20 and voltage mapping with respiration correction will be performed at such a density that the color threshold will be 10 mm. Additional mapping with an ablation catheter will be recommended at sites that are difficult to record with a multipolar 1 mm electrode catheter. When mapping with an ablation catheter, contact force ≥3 g will be required. An LVA will be defined as <0.5 mV. Patients with a total of LVA ≥5 cm^2^ will be randomized to either LVA guided ablation in addition to PVI or PVI alone. Patients with a total area of LVAs <5 cm^2^ will be excluded.

#### 2.4.3 Low‐voltage area guided ablation

Cluster ablation for the whole LVA will be performed in patients allocated to LVA guided ablation in addition to PVI. LVAs in the posterior wall may be isolated by linear ablation to avoid esophageal injury. The Visitag module will be used with the following settings: catheter stability ≤2 mm for 5 s, contact force ≥5 g (time ≥ 25%), tag size 6 mm in diameter. Radiofrequency will be delivered to the LVA until an AI of ≥350 and the distance between the tags will be <6 mm. At the discretion of the physician, avoidance of ablation near Bachmann's bundle or near the His bundle will be acceptable if there are concerns they will be injured. The area of residual LVAs that are not ablated will be calculated. Scar areas are not included in the residual LVA.

#### 2.4.4 Procedure after low‐voltage area guided ablation or PVI

Following LVA guided ablation in addition to PVI or PVI alone, tachyarrhythmia inducibility will be evaluated using 10‐s burst pacing from the coronary sinus and right atrium at the maximum current output (20 mA), beginning with a cycle length of 400 ms and decreasing to 200 ms. Isoproterenol administration will be used to evoke non‐PV AF triggers. Physicians will perform focal ablation of AF triggers or isolate AF triggers, and ablate to block the cavotricuspid isthmus (CTI) in cases with inducible CTI‐dependent atrial flutter (AFL). Ablation for clinical coexisting tachyarrhythmias such as AFL, atrial tachycardia, and supraventricular tachycardia will also be performed. Physicians will confirm PVI stability by checking the disappearance of the potential in the PV > 20 min after the initial PVI and finish the procedure.

#### 2.4.5 Antiarrhythmic drugs and repeat ablation after the ablation

The blanking period after the protocol therapy will be 3 months. AF during this period will not be considered as a recurrence. Anti‐arrhythmic drugs will be strongly recommended not to use after 3 months of the ablation.

Repeat ablation will be performed for recurrent of AF at the discretion of the investigators. During repeat ablation procedures, reisolation of reconnections to the PV and voltage mapping after reisolation of the PV will be required. In patients with PVI alone, LVA guided ablation will not be performed. In patients with LVA guided ablation in addition to PVI, repeat LVA guided ablation may be performed. In patients in both groups, ablation other than LVA guided ablation including linear ablation can be performed at the discretion of the physician.

#### 2.4.6 Collection of clinical and follow‐up data

Before ablation, baseline data, information on AF, previous history, and medication history will be collected, and blood tests, transthoracic echocardiography, and 12‐lead ECG will be acquired. Detailed data on the first ablation procedure will be collected. A medication assessment and 12‐lead ECG will be performed at discharge. Adverse events will be collected from the first procedure to 6 months after the first ablation procedure.

#### 2.4.7 Follow‐up schedule

Patients will be followed at 3, 6, and 12 months after ablation. A 12‐lead ECG, Holter ECG, medication assessment, and blood tests will be acquired at 6 and 12 months. Transthoracic echocardiography will be performed at 12 months. Twice‐daily 30 s rhythm checks and symptom‐driven checks will be performed with portable ECG from 6–12 months.

#### 2.4.8 Study endpoints

The primary endpoint of the study will be the recurrence of AF documented by scheduled or symptom‐driven ECG tests during the 1 year follow‐up period after the index ablation procedure. “Recurrence of AF” is defined as the documentation of any atrial arrhythmia including AF, AFL, and/or AT lasting ≥30 s by 12‐lead ECG or other appropriate tests. The endpoint will be determined by the endpoint committee, which will be composed independently of protocol developers, data and safety monitoring board, clinical research funders, investigators, subcontractors, and collaborators.

Secondary endpoints will include death from any cause, symptomatic cerebrovascular stroke, bleeding events, recurrent AF after repeat ablations, and any periprocedural adverse events. Bleeding events are defined as major bleeding in the ISTH bleeding criteria^17^ and bleedings needing for hospitalization.

### 2.5 Study organization and status

#### 2.5.1 Osaka Cardiovascular Conference

The Osaka Cardiovascular Conference (OCVC) consists of cardiologists belonging to the Osaka University Graduate School of Medicine or one of its 30 affiliated Osaka hospitals. The OCVC was initiated in 2014 to investigate clinical questions in the realm of cardiovascular medicine. Among participating institutions, physicians from seven hospitals that perform a large volume of invasive treatments for AF comprise the OCVC‐SUPPRESS‐AF investigators and are participating in this study.

SUPPRESS‐AF started enrollment in June 2019, and completion of enrollment is expected in March 2022. Completion of the study is expected by March 2023.

#### 2.5.2 Statistical considerations

The sample size and randomization were formulated to achieve the primary objective. That the primary outcome's incidence rate in the PVI alone group will be the same as the rate in the LVA guided ablation in addition to PVI group is the null hypothesis. The recurrence rate of AF is assumed to be 40% in the PVI alone group and 25% in the LVA guided ablation plus PVI group. Thus, a sample size of 155 subjects in each group will be required to reject the null hypothesis with a power of 80% and a significance level of 5%. With a randomization ratio of 1:1, we will need a total sample size of 340 patients, allowing for some dropouts.

The full analysis set (FAS) is the set of all randomized patients meeting all inclusion criteria, and its statistical evaluation uses the intention‐to‐treat principle: patients should be analyzed according to the treatment assigned at randomization. The per protocol set (PPS) will be the set of patients in the FAS who complete the study without protocol violations.

Analysis of the primary outcome in each group will be estimated by the Kaplan–Meier method for FAS and PPS. The 12‐month recurrence rate and its 95% confidence interval after initial ablation will be calculated using Greenwood's formula, and the log‐rank test will be used for comparison between the two groups. No adjustment for multiplicity will be required because of the closed testing procedure. No interim analyses will be conducted in this trial.

## 3 SUMMARY

The SUPPRESS‐AF trial will be a prospective, randomized, multicenter, open‐label trial for persistent AF patients undergoing their first catheter ablation. The effects of LVA guided ablation in addition to PVI compared to PVI alone on the incidence of recurrent AF documented by scheduled or symptom‐driven ECGs during the 1 year follow‐up period after the index ablation will be compared. The results will reveal whether LVA guided ablation in addition to PVI is superior to PVI alone to maintain sinus rhythm, and will provide additional data to develop treatment strategies for persistent AF.

## CONFLICT OF INTEREST

Masaharu Masuda is an advisory board of Boston and has received speaker honoraria from Biosense Webster, Inc., Abbott, Boston and Medtronic, payment for a manuscript from Boston and Medtronic, and research grants from Biosense Webster, Inc. and Medtronic. Koichi Inoue has received speaker honoraria from Biosense Webster, Inc. and Medtronic. Nobuaki Tanaka has received speaker honoraria from Biosense Webster, Inc. and Medtronic. Tetsuya Watanabe has received speaker honoraria from Medtronic and Abbott. Yoshio Furukawa has received speaker honoraria from Biosense Webster, Inc., Abbott, Boston and Medtronic. Yasuyuki Egami has received speaker honoraria from Biosense Webster, Inc. and Boston and research grants from Abbott, Boston and Japan Lifeline. Akio Hirata has received speaker honoraria from Abbott. Hitoshi Minamigushi is an advisory board of Medtronic, Abbott and Japan Lifeline, has received speaker honoraria from Medtronic, Abbott, Japan Lifeline, Nihon Kohden and Biotronik, and payment for a manuscript from Medtronic and Abbott. Takafumi Oka has received speaker honoraria from Medtronic, Abbott, Boehringer Ingelheim, Bayer, Bristol‐Myers Squibb, Daiichi Sankyo, AstraZeneca, Biotronik, GE health care and Japan Lifeline. Yohei Sotomi has received research grants and speaker honoraria from Abbott Vascular Japan, Boston Scientific Japan, Terumo, Japan Lifeline, Biosensors, Medtronic, Daiichi‐Sankyo, Bayer, Boehringer Ingelheim, and Bristol‐Myers Squibb and is an endowed chair funded by Terumo, Asahi Intecc, Nipro, and Shimadzu Corporation. Hiroya Mizuno has received speaker honoraria from Abbott, Medtronic and Johnson & Johnson, and is endowed chair funded by Terumo, Nipro, Asahi Intecc and Shimadzu Corporation. Shungo Hikoso has received research grants from Abbott, Biosense Webster, Inc. and Medtronic. Yasushi Sakata has received speaker honoraria from Abbott, Boston and Medtronic, and scholarship donations from Abbott, Boston and Medtronic. The other authors have no conflicts of interest to report.

## Data Availability

Author elects to not share data.

## Appendix A.

The OCVC‐SUPPRESS‐AF Investigators:

Shinji Hasegawa and Miwa Miyoshi: Osaka Hospital, Japan Community Healthcare Organization Osaka, Japan; Toshiaki Mano, Masaharu Masuda and Takashi Kanda: Kansai Rosai Hospital, Amagasaki, Japan; Masatake Fukunami, Takahisa Yamada, Tetsuya Watanabe, Yoshio Furukawa and Masato Kawasaki: Osaka General Medical Center, Osaka, Japan; Yoshiharu Higuchi, Akio Hirata, Nobuhiko Makino and Hitoshi Minamiguchi: Osaka Police Hospital, Osaka, Japan; Jun Tanouchi, Masami Nishino, and Yasuyuki Egami: Osaka Rosai Hospital, Sakai, Japan; Yasushi Sakata, Yasushi Matsumura, Shungo Hikoso, Daisaku Nakatani, Shinichiro Suna, Hiroya Mizuno, Toshihiro Takeda, Tetsuhisa Kitamura, Takafumi Oka, Katsuki Okada, Tomoharu Dohi, Kentaro Ozu, Tomoaki Nakano, Yohei Sotomi, Akihiro Sunaga, Taiki Sato, Hirota Kida, and Oeun Bolrathanak: Osaka University Graduate School of Medicine, Suita, Japan; Kenshi Fujii, Katsuomi Iwakura, Koichi Inoue, and Nobuaki Tanaka: Sakurabashi Watanabe Hospital, Osaka, Japan; and Shiro Hoshida and Tomoko Minamisaka: Yao Municipal Hospital, Yao, Japan.
